# Shells of *Littorina littorea* (Gastropoda)—an archive for palaeoclimate and seasonal shellfish collection in medieval and early modern Orkney?

**DOI:** 10.1098/rstb.2024.0039

**Published:** 2025-07-10

**Authors:** Bernd R. Schöne, Holly Young, Hubert Vonhof, Jennifer Harland

**Affiliations:** ^1^ Institute of Geosciences, University of Mainz, Mainz 55128, Germany; ^2^ Archaeology Institute, University of the Highlands and Islands Orkney College, Kirkwall, Orkney KW15 1LX, UK; ^3^ Climate Geochemistry Department, Max Planck Institute for Chemistry, Mainz 55128, Germany; ^4^ Department of Archaeology, University of York, York, Yorkshire YO10 5DD, UK

**Keywords:** sclerochronology, climate normal, oxygen-isotope-derived water temperature, vital effect, shell midden archaeology

## Abstract

*Littorina littorea—*the common periwinkle*—*is biogeographically widely distributed and occurs frequently in archaeological shell middens along the European Atlantic coast. Yet, its potential use as a recorder of palaeoclimate and the season-of-collection has not been rigorously assessed. Here, gaps of knowledge are addressed with a comprehensive dataset of 78 specimens collected from Skaill Farm, a Medieval and Post-medieval archaeological site on the island of Rousay, Orkney, UK, and the adjacent intertidal zone, and more than 3000 shell oxygen isotope data. Shell *δ*^18^*O*-based coastal summer water temperature deviated by only +0.18°C from the 1992/93–2022 average, but winter temperatures were overestimated by 2–5°C, provided proper sampling was applied. Modern summers are 0.7°C warmer than during the final centuries of the late Little Ice Age, but statistically indistinguishable from such during two Medieval intervals (14th–15th century). As the timing and rate of seasonal shell growth remains unchanged through the lifetime, the season of harvest can be faithfully determined from apertural *δ*^18^*O* profiles. During studied time intervals in the past, snails were predominantly collected in early spring and possibly functioned as a starvation food. Findings of this study may encourage future shell midden archaeological studies at sites where the common periwinkle occurs.

This article is part of the theme issue ‘Shifting seas: understanding deep-time human impacts on marine ecosystems’.

## 1. Introduction

The common periwinkle, *Littorina littorea* (Linnaeus, 1768) natively inhabits the rocky intertidal, salt marshes and shallow subtidal between the White Sea and Gibraltar, including the British Isles, the Baltic Sea and Spitsbergen [1,2]. It also occurs as an invasive species (confirmed by genetic evidence [3]) in the Mediterranean (Turkey) and Australia, as well as the Atlantic and Pacific coasts of North America [2,4–6]. Since it is an edible gastropod, it can be frequently found in archaeological shell middens along the European Atlantic coastline [7], specifically Spain [8], Portugal [9], Denmark [1,10–12] and the UK [1,13–16]. Notable archaeological finds outside Europe comprise Nova Scotia (three Mi’kmaw camp sites near Halifax, *ca* 1300 yr CE [17]) and Newfoundland (Norse settlements, *ca* 1000 yr CE [18]). The oldest known fossil specimens are from the Pliocene of Iceland (Tjörnes Beds [19]) and Suffolk, UK (Red Crag Formation [1,20,21]). As the outer layer of the shell consists of low-Mg calcite (while the inner is aragonitic [22]), it should be possible to derive pristine environmental signals from the light stable isotope values of fossils and precisely reconstruct the season of collection from archaeological material.

In view of the large biogeographic distribution of *L. littorea*, its rich fossil record and ubiquitous occurrence in kitchen waste mounds, surprisingly little sclerochronological and shell midden archaeological work exists compared with other gastropod taxa such as *Patella* spp. [23–27] and *Phorcus* spp. [14,28–33]. Some key findings shall be briefly summarized here. Ekaratne & Crisp [34] identified distinct tidal growth patterns in the shell that might be useful for precise seasonal alignment purposes. Prior to sexual maturity, the common periwinkle reportedly grows uninterruptedly during both seasonal extremes and thus preserves information on the full seasonal temperature amplitude in the oxygen isotope values of its shell (*δ*^18^*O*_shell_) [35], provided that water temperature remains above the lower growth threshold of 3.7 °C [36]. As these authors further explained, at localities where winter temperatures regularly fall below this critical threshold, the difference between expected and measured *δ*^18^*O*_shell_ values can be used to quantify changes in winter salinity, a tool that was recently employed to investigate the causes for the faunal turnover (i.e. oyster decline; rise of cockles and mussels) during the Mesolithic–Neolithic transition in Denmark [37]. Following Burman & Schmitz [36], shell formation occurs with a constant *δ*^18^*O* offset of +2 ‰ from expected equilibrium fractionation. Taking this offset in mind, Burman & Påsse [38] demonstrated that summer temperatures during the Eemian were 1 to 3°C higher than today in the Kattegat and English Channel. Shell oxygen isotope profiles of the common periwinkle from shell middens were also used to infer the season of collection [37,39], which can provide insights into subsistence strategies of past human societies.

Despite these interesting and relevant findings, the life history traits of *L. littorea* remain largely unknown because existing sclerochronological studies focused exclusively on the juvenile portions of the shells, for the not entirely unjustified reason that only those could provide information on the actual environmental seasonality [35,36,38]. Also, inconsistencies exist between sclerochronological data [34,36] and behavioural studies in the field and laboratory regarding the physiological activity during winter [40], which has implication for the lower shutdown temperature of shell growth. Furthermore, the number of studied specimens and annual increments remained very limited in existing sclerochronological studies (e.g. Burman & Påsse [38]: 2 to 3 shells from a single site and time interval, 1 to 3 years per specimen). This aggravates the assessment of the statistical robustness of the data (season-of-collection, differences between palaeoenvironmental data reconstructed from contemporaneous specimens, etc.). While a remarkable temporal resolution of about 2 weeks or so was achieved in the above-cited works, neither of these datasets can technically be used to reconstruct climate because none of them meet the definition of climate, which is the average and variability of weather over a minimum of 30 consecutive years [41,42]. Admittedly, only few mollusc sclerochronological studies fulfill this requirement, particularly those dealing with specimens that barely live for more than a decade.

The primary goal of the present study therefore was to increase our knowledge on the timing and rate of seasonal shell growth—including ontogenetic changes thereof, to evaluate strategies and pitfalls of isotope sampling strategies and explore the scope for palaeoclimate and season-of-collection reconstructions based on *L. littorea*. Based on extensive autecological work on the common periwinkle [40], we hypothesize that it would be nearly impossible to obtain faithful information on environmental conditions that prevailed during winter, not even in year one. However, we assume that it should be possible to infer summer temperatures reliably even from old-grown specimens. Aside from live-collected specimens, 64 shells from three different stratigraphic horizons (Medieval to late Little Ice Age (Post-medieval); *ca* 14th−19th century) of shell midden deposits at Skaill Farm, Rousay, Orkney, Scotland were used to reconstruct climate (water temperature) and briefly assess changes of the season-of-harvest during the past millennium. Results of the present study can potentially serve as an encouragement to intensify sclerochronological studies of periwinkles recovered from archaeological contexts.

## 2. Material and methods

### (a) Archaeological context

**Figure 1.**
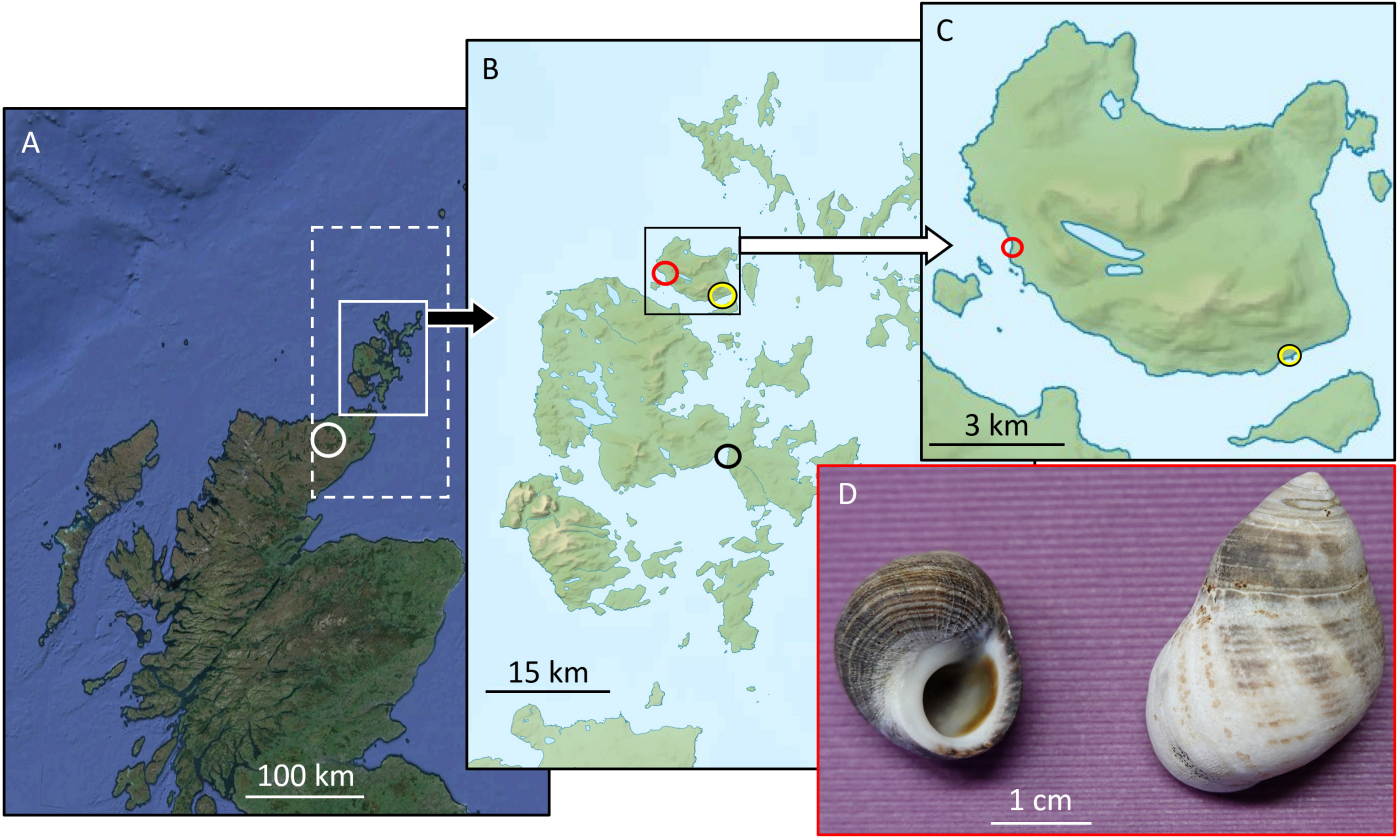
Map showing sampling localities (A–C) and examples of *Littorina littorea* ((D); left: modern specimen A3; right: Medieval 2 specimen 144-D1). (A) Overview map of northern Scotland. Dashed rectangle = source of NOAA ERSST data (58°N, 002°W–60°N, 004°W). White circle = *δ*^18^*O* data in precipitation from GNIP station Altnabreac (58°23'00.24"N, 003°42'01.08"W, 155 m asl). (B) Magnified solid white rectangle in (A): Orkney. Black circle = Scapa Pier monitoring site (temperature, salinity). Yellow circle = Rousay Pier, where water samples for *δ*^18^*O*_water_ (and δD) analyses were collected biweekly. Red circle = Skaill Farm, Westness, Rousay, where modern and archaeological common periwinkles were collected. (C) Magnified solid black rectangle in (B): Localities on Rousay where water samples (yellow circle: Rousay Pier) and shells (red circle: Skaill Farm) were collected. Maps to the right and in the middle were modified after the map ‘Orkney Islands UK relief location map.jpg’ generated by user Nilfanion using Ordnance Survey data, CC BY-SA 3.0, downloaded at https://de.m.wikipedia.org/wiki/Datei. Satellite image in (A) is modified after Google Earth (Data SIO, NOAA, U.S. Navy, NGA, GEBCO, Image Landsat/Copernicus).

Despite its comparatively small size (49 km^2^), the island of Rousay is remarkably rich in archaeological sites [43] including Neolithic tombs, Iron Age brochs, Viking and Medieval settlements and cemeteries, up to and including Orkney’s only ‘Highland’ clearance landscape [44]. The archaeology of the island has thus been extensively studied since the early 20th century. In the present study, the focus was placed on Skaill farmstead, located close to the WSW shore of the island of Rousay, Orkney, *ca* 1 km NNW of Westness (figure 1). It consists of upstanding Post-medieval farm buildings (NMRS site number HY33SE 161), Late Norse, Medieval and Post-medieval structures, and associated pottery-bearing kitchen midden deposits containing remnants of fish, mammal and bird bone, and shellfish (specifically, shells of patelloids, mussels and common periwinkles). Ongoing excavations since 2015, run by the University of the Highlands and Islands, indicate the site has a Viking/Norse foundation directly below the upstanding remains, while ^14^C dating and stratigraphical sequences suggest continuous occupation for at least 1000 years. The area was cleared in the 19th century by landlords, thus inadvertently preserving the archaeology through lack of modernization and development.

### (b) Environmental datasets

Daily sea surface temperature (SST) and weekly sea surface salinity (SSS) data of the Scapa Pier Monitoring site (58°57'25.20"N, 002°58'22.19"W, black circle in figure 1B)—the closest locality to Skaill Farm (red circles, figure 1B,C) providing environmental data from the coastline—were obtained from the website of the Scottish Government (https://data.marine.gov.scot; last access 16 May 2024). These data covered the time between November 1999 and January 2021 (SST) and 20 December 2020 (SSS), respectively. Gaps in the temperature record were filled with data obtained from a linear model constructed from the Scapa Pier SST and remotely sensed SST (58°N, 002°W–60°N, 004°W; figure 1; NOAA Extended Reconstructed SST (ERSST) v. 5 [45], monthly mean subset, obtained from NOAA/ESRL/PSD at their website https://www.esrl.noaa.gov/psd/, last access 16 May 2024; daily ERSST were inferred by linear imputation). This model was also used to interpolate SST since the end of the monitoring programme (early 2021). Weekly SSS data ( = once measurement per week; 2000−2020) were converted to daily resolution by linear imputation. These data were then used to compute long-term daily salinity and fill the gap between 20 December 2020 and the date of collection of the living periwinkles (14 July 2022).

Twenty-three water samples were taken on a nearly biweekly basis at Rousay Pier (yellow circle, figure 1) during 2022, i.e. during the year of collection of living *L. Littorina* near Skaill Farm (figure 1), and isotopically characterized (electronic supplementary material). With these data, a numerical model ( = local freshwater mixing line) was computed to infer *δ*^18^*O*_water_ values from salinity data (electronic supplementary material). For the *y*-intercept (0 practical salinity units (PSU)), we used the arithmetic average (−7.99 ‰) of 13 *δ*^18^*O* data in precipitation (1981−1982) measured at the station Altnabreac (58°23'00.24"N, 003°42'01.08"W, 155 m asl; obtained from the IAEA/WMO—Global Network of Isotopes in Precipitation, GNIP database at https://nucleus.iaea.org/wiser, last access: 17 May 2024). Following this model, a change in salinity by 1 PSU results in a change of *δ*^18^*O*_water_ by 0.23 ‰. The long-term seasonal salinity fluctuation of 34.46 ± 0.52 PSU (average ± 1 *σ*) (range: 27.75 to 35.16 PSU) thus translates into a *δ*^18^*O*_water_ average of −0.20 ± 0.12‰ (range: −0.04 to −1.71 ‰). The average *δ*^18^*O*_water_ value was later used to compute temperature from *δ*^18^*O*_shell_. For comparison, we also reconstructed temperature from *δ*^18^*O*_shell_ using daily imputed salinity data.

### (c) Common periwinkle samples

For calibration purposes, seven specimens of *L. littorea* (A1 to A7; table 1) were collected alive—without preference for size—on 14 July 2022 within the mid-intertidal zone adjacent to Skaill Farm (59°09'12"N, 003°05'50"W; red circles in figure 1). Soon after collection, specimens were placed in near-boiling water (*ca* 90°C) for several seconds. This treatment facilitated removal of the soft parts. Shells were then rinsed in tap water and adhering algae were scrubbed off with a tooth brush; thereafter shells were air-dried. Five shells were arbitrarily selected for stable oxygen (and carbon) isotope analyses and the remaining two for the study of the inner structure (growth patterns) and mineralogy of the shells (table 1). For this purpose, a hand-held drill equipped with a wafer-thin diamond disk was used to cut a *ca* 1.5 cm long and 3 mm broad section from the aperture of both shells, perpendicular to the major (annual) growth lines visible on the outer shell surface (OSS) along the maximum growth axis. One side of each cross-section was ground on 800 and 1200 grit paper and polished with 2000 grit paper. The polished shell slabs were then studied under a binocular microscope. In particular, we were interested in the internal growth patterns (electronic supplementary material, figure S1+2), their geometry relative to the OSS and the spatial distribution, thickness and arrangement of the calcitic outer shell layer (OSL; irregular prismatic ultrastructure) and the aragonitic inner shell layer (ISL; crossed-lamellar ultrastructure [22]) (electronic supplementary material, figure S2). The angle at which the growth lines reached the OSS was used to inform the isotope sampling strategy (electronic supplementary material, figure S2). Since most isotope sampling was accomplished on the OSS by micromilling (see §2d), the time-averaging of each sample and the suitable distance between sampling positions were defined by the milling depth in relation to the growth pattern geometry. As illustrated by the sketch in electronic supplementary material, figure S2, for a given milling depth, samples cover more time if the growth lines reach the OSS with a flat angle than with a steep angle. In the latter case, much higher temporal resolution can be achieved, because the distance between milling swaths can be reduced without risking temporal overlap of samples.

**Table 1.** Overview of shell samples (*Littorina littorea*) used in present study and their purpose of use. Specimens used for climate reconstruction (= water temperature) and labelled with ‘oip’ were sampled from the apex to the aperture, i.e. ontogenetic isotope profiles were generated. SoC = season of collection. Numbers in curly brackets denote number of specimens. ‘Internal structure’ includes analysis of different shell layers, growth patterns and growth pattern geometry. Listed calibrated radiocarbon ages: 95% confidence interval; for details see electronic supplementary material.

time	specimen ID	isotopes					lifespan (yr)	apex-to-aperture length	unrolled length	internal structure	mineralogy (Feigl)
		*N* total	OSS mm	apertural mm	(palaeo)climate	SoC					
	**↓*n* = 78; *N* →**	**3013**	**2898**	**107**	**51, (oip: 20)**	**64**	**20**	**46**	**20**	**2**	**1**
modern	A1	151	134	17	X (oip)		X	X	X		
	A2	165	137	28	X (oip)		X	X	X		
	A3	114	113	1	X (oip)		X	X	X		
	A4	152	150	2	X (oip)		X	X	X		
	A5	141	141		X (oip)		X	X	X		
	A6									X	X
	A7									X	
late LIA (late 17th – 19th century CE) 1683−1936 cal yr CE	133-D1	65	55	10	X (oip)	X	X	X	X		
	133-D2	83	78	5	X (oip)	X	X	X	X		
	133-D3	75	70	5	X (oip)	X	X	X	X		
	133-D4	93	93		X (oip)	X	X	X	X		
	133-D5	112	105	7	X (oip)	X	X	X	X		
	133-D… {5}	38	38		X {1}	X {2}		X {5}			
Medieval 2 (early 14th – early 15th century CE) 1308−1424 cal yr CE	144-D1	85	85		X (oip)	X	X	X	X		
	144-D2	92	84	8	X (oip)	X	X	X	X		
	144-D3	65	60	5	X (oip)	X	X	X	X		
	144-D4	90	90		X (oip)	X	X	X	X		
	144-D5	122	122		X (oip)	X	X	X	X		
	144-D… {22}	355	355		X [9]	X {22}		X {3}			
Medieval 1 probably early 14th–early 15th century CE	412-D1	109	109		X (oip)	X	X	X	X		
	412-D2	88	78	10	X (oip)	X	X	X	X		
	412-D3	80	75	5	X (oip)	X	X	X	X		
	412-D5	85	85		X (oip)	X	X	X	X		
	412-D6	77	75	2	X (oip)	X	X	X	X		
	412-D… {29}	576	576		X {21}	X {25}		X {18}			

**Figure 2.**
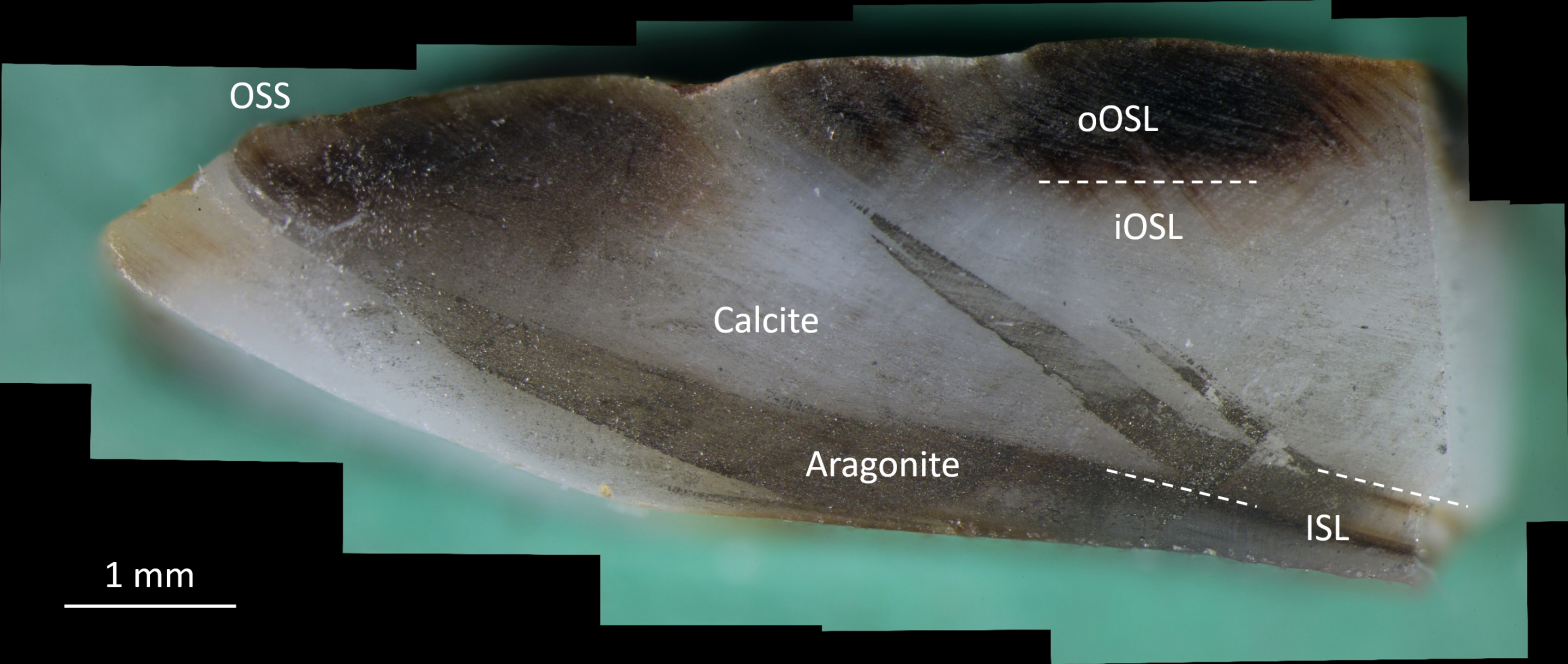
Polished cross-section of modern *Littorina littorea* (specimen A6, collected alive near Skaill Farm on 14 July 2022) 5 min after treatment with Feigl solution. Aragonite became dark grey to black, but calcite only light grey owing to differential dissolution speed. Note, during some winters, the aragonitic inner shell layer (ISL) stretches up to the outer shell surface (OSS), e.g. in last winter prior to collection. The inner shell layer (ISL) consists of aragonite (black after staining), whereas both sublayers of the outer shell layer (oOSL and iOSL) are made of calcite (light grey after staining; brown colour in the oOSL stems from carotinoid pigments). During winter, when growth rate slows down, the dark stained aragonitic inner shell layer can reach the outer shell surface (OSS). Low-Mg calcite samples taken from the shell by means of OSS micromilling (see electronic supplementary material, figure S2) would then be contaminated with a fraction of aragonite, which would lead to underestimation of water temperature if this remained unnoticed (or unconsidered) and the palaeothermometry equation for low-Mg calcite was used. Shell growth is from right to left. Winter lines typically form in January/February, *ca* a month before the seasonal temperature minimum. o/iOSL = outer/inner portion of the outer shell layer.

One of the polished sections was also used to map the mineralogy. Therefore, two droplets of Feigl solution were placed on its surface and images taken at 30 s intervals (figure 2). Feigl solution can be used to visually distinguish aragonite from calcite because the former dissolves more quickly in water than the latter and assumes a darker grey tint than calcite when immersed in Feigl solution for a few minutes [46,47]. Dissolution of CaCO_3_ is associated with the release of OH– ions that combine with Mn and Ag of the Feigl solution to form (black-coloured) oxides.

Archaeological shells were obtained from different stratigraphic horizons within the middens at Skaill Farm, from contexts 412 (‘Medieval 1’, probably early 14th to early 15th century CE), 144 (‘Medieval 2’, early 14th to early 15th century CE) and 133 (‘Post-medieval’ = ‘late Little Ice Age’ (LIA), late 17th to 19th century CE) (electronic supplementary material, figure S3). Absolute ages of the layers were determined by radiocarbon dating of terrestrial mammal remains, augmented by relative dating provided by artefactual analysis (electronic supplementary material). All fossil shells were much more brittle than modern ones and required a gentler cleaning procedure prior to sampling for isotopes. A glass fibre pencil was used to remove any adhering sediment and occasionally occurring glauconite grains. Archaeological shell were used to assess how climate in the study region changed during the past millennium and to determine the season when the snails were collected.

### (d) Isotope sampling and analysis

**Figure 3.**
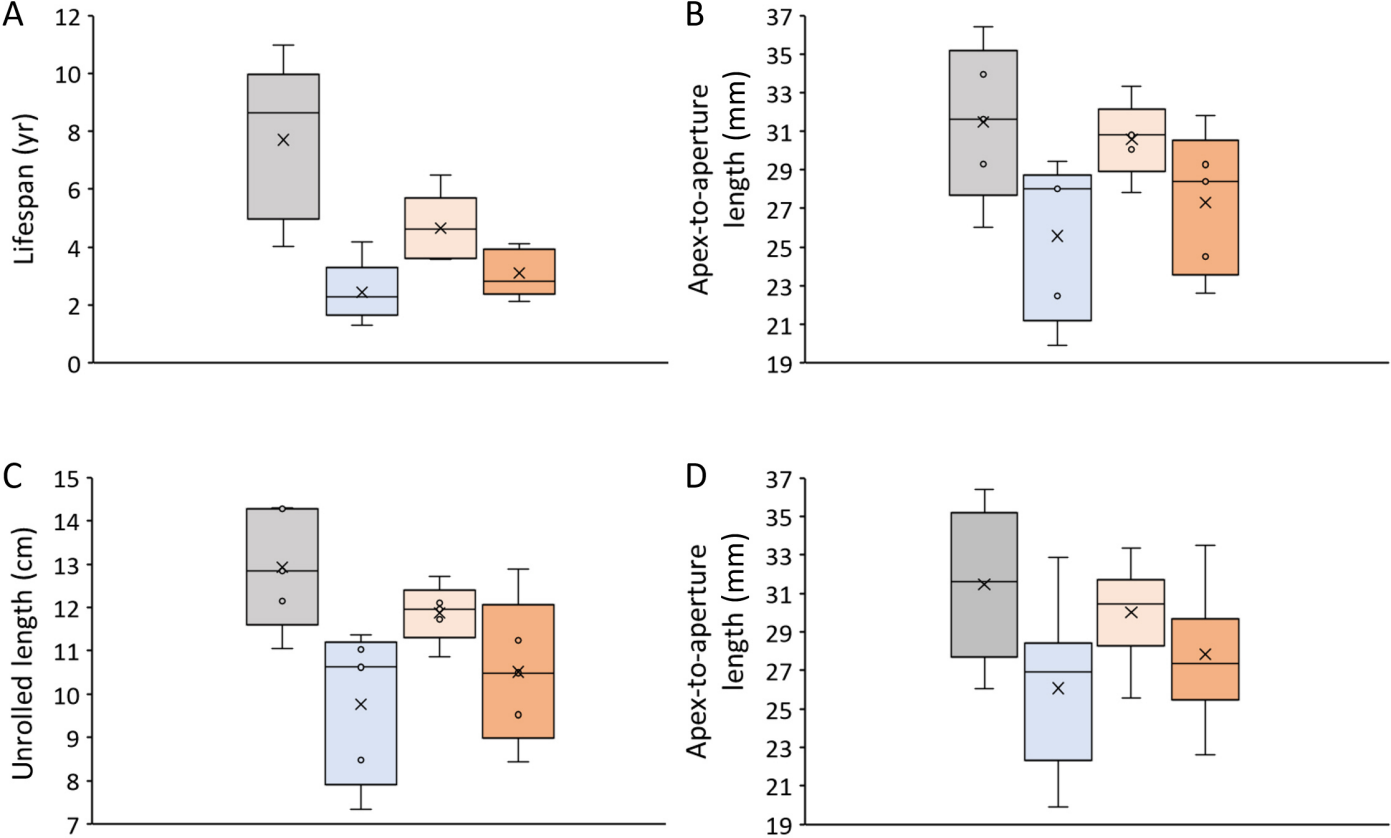
Size and ontogenetic age data of the studied *Littorina littorea* specimens including pairwise statistical comparison (Mann–Whitney *U*). Colour code: Light grey = modern specimens; light blue = Post-medieval (late LIA); light orange = Medieval 2; darker orange = Medieval 1. (A–C) 20 specimens, five per time interval, that were used in electronic supplementary material, figure S5; (D) 26 additional archaeological specimens (five from the late LIA, three from Medieval 2, 18 from Medieval 1). (A) Lifespan, (B,D) apex-to-aperture length, (C) Unrolled length (measured along axis of maximum growth from apex to aperture). See also table 2 for pairwise statistical comparison and more details on number of samples per dataset.

Most of the isotope sampling was done by hand on the OSS (along the axis of maximum growth) using a micromilling device firmly attached to a binocular microscope and equipped with a conically shaped SiC (widia) drill bit (Komet/Gebr. Brasseler GmbH & Co. KG, model no. H52 104 003; tip diameter 300 µm). Shell carbonate powder (*ca* 90 ± 30 µg; *n* = 2908 samples; see list in electronic supplementary material) was obtained from the outer portion of the outer shell layer (oOSL) subparallel to the shape of the growth lines. Along the first whorl, sample swaths gradually became longer (parallel to the growth lines) and narrower (perpendicular to the growth lines), the milling depth quintupled and the distance between millings decreased, i.e. near the apex, milling swaths measured approximately 2000 × 2000 × 10 µm (length × width × depth) and reached approximately 3500 × 300 × 50 µm close to completion of the first whorl; the latter OSS milling dimensions were maintained until the aperture (electronic supplementary material, figure S4+5). All five modern and five arbitrarily selected fossil specimens of each of the three archaeological layers (i.e. a total of 20 shells) were sampled from the apex to the aperture by surface micromilling to generate ontogenetic isotope curves covering the entire lifespan of the specimens (electronic supplementary material). In these 20 specimens, the distance between the centres of the OSS micromilling swaths, the micromilling widths and the width of unsampled shell portions (if present) were digitally measured. In conjunction with the temporally aligned isotope data (electronic supplementary material, figure S6), these data allowed us to compute the timing and rate of seasonal shell growth (electronic supplementary material, figure S7). In this context, we also determined the age of the specimens (figure 3A) and the apex–aperture length (with a digital caliper) (figure 3B,D), as well as in digital images, the unrolled (unfolded) shell length (figure 3C) and the widths of the annual increments to assess cumulative annual shell growth curves (electronic supplementary material, figure S8). From the remaining fossils, only the last centimetre or so at the aperture was sampled by OSS micromilling to determine the season of death. Often, these data covered more than a year of growth and could then be used to assess seasonal temperature variations.

In some specimens, a few samples (*n* = 107) were also taken from the inner shell surface (ISS) at the aperture (backward in the direction to the apex) to obtain a more highly resolved isotope record (electronic supplementary material, figure S9). In specimen A2, the entire last annual increment was sampled with this apertural micromilling technique. As in the case of OSS micromilling, milling widths were determined during apertural micromilling to determine the timing and rate of seasonal growth.

Shell powder samples (*ca* 50–120 µg) were dissolved in water-free phosphoric acid at 72°C in He-flushed borosilicate exetainers (12 ml). Generated CO_2_ gas was measured with a Thermo Fisher Scientific MAT 253 isotope ratio mass spectrometer operated in continuous flow mode and coupled to a GasBench II. Isotope data were calibrated against a Carrara marble standard (IVA Analysentechnik GmbH & Co. KG, Meerbusch, Germany). Results were presented in δ-notation relative to the Vienna Pee Dee Belemnite (V-PDB).

The isotope values of the seawater samples were measured at the Max Planck Institute for Chemistry by isotope ratio infrared spectroscopy. A volume of 0.5 µl from each water sample was injected into a moisturized nitrogen carrier gas and subsequently analysed on a Picarro L2140-i water isotope analyser [48]. The use of a moisturized carrier gas removes memory effects, and the requirement of only 0.5 µl of water for a precise analysis minimizes the salt build-up in the injection port of this system, allowing for longer series of seawater samples to be run before cleaning of the injection unit is required. Two lab-internal water standards calibrated to the V-SMOW-SLAP scale (MainzTap2022: *δ*^18^*O*_water_ = −8.75‰ and δ^2^H_water_ = −63.53‰; and Kona2022: *δ*^18^*O*_water_ = 1.51 ‰ and δ^2^H_water_ = 3.48‰) were routinely analysed in this series and used for conversion of the isotope data of the seawater samples to the V-SMOW-SLAP scale. When each injection is treated as an individual analysis, the precision (1 SD) of 10 replicates of each of these standards, run interspaced with the samples, is 0.10‰ for *δ*^18^*O*_water_ (and 0.50‰ for δ^2^H_water_). Salt loading in the injector port and microsyringe likely leads to somewhat increased analytical uncertainty of the seawater samples in comparison with the (freshwater) isotope standards. Based on sample replication data, we estimate a realistic single-injection uncertainty for the isotope analysis of seawater samples to be 0.15‰ for *δ*^18^*O*_water_ (and 0.60‰ for δ^2^H_water_).

### (e) Temporal alignment, growth rate and season of collection

To place the shell record in precise temporal context, reconstructed water temperature data were compared with the observed water temperature curve. For this purpose, temperature (T*δ*^18^*O*) was computed from *δ*^18^*O*_shell_ values using the palaeothermometry equation by Hays & Grossman [49]:


(2.1) $$T_{\delta^{18}O}=15.7-4.36\cdot \left(\delta^{18}O_{shell}-\delta^{18}O_{water}\right)+0.12\cdot {\left(\delta^{18}O_{shell}-\delta^{18}O_{water}\right)}^{2}$$

where *δ*^18^*O*_shell_ is given versus V-PDB and *δ*^18^*O*_water_ versus V-SMOW. We assumed a constant *δ*^18^*O*_water_ value of −0.20 ± 0.12‰ through seasons and years. As outlined further above, this value represents the long-term average reconstructed from salinity and is nearly identical to the average of measured *δ*^18^*O*_water_ values in 2022 (−0.19 ± 0.15‰; electronic supplementary material). T*δ*^18^*O* data were then aligned with the instrumental temperature curve to achieve the best fit with the (sinusoidal) shape of the seasonal temperature oscillations (electronic supplementary material, figure S6A). The isotope records of fossil shells were aligned in the same way, i.e. using the modern *δ*^18^*O*_water_ value and the long-term (2000−2020) temperature curve (electronic supplementary material, figure S6B–D).

It should also be added that the same alignment result was achieved by computing a pseudo-*δ*^18^*O*_shell_ chronology from instrumental temperature and measured/reconstructed *δ*^18^*O*_water_ data. This approach should be used if the *δ*^18^*O*_water_ value (and hence salinity) fluctuates strongly during seasons and years to avoid the need to iteratively re-compute T*δ*^18^*O* values with actual *δ*^18^*O*_water_ values until the best alignment is found. Similar results can also be achieved by using the approach of Fenger *et al.* [50], which is based on the fractionation equation (1000 ln *α*) for low-Mg calcite and water by Friedman & O’Neill [51], on which the quadratic approximation by Hays & Grossman [49] is based.

**Figure 4.**
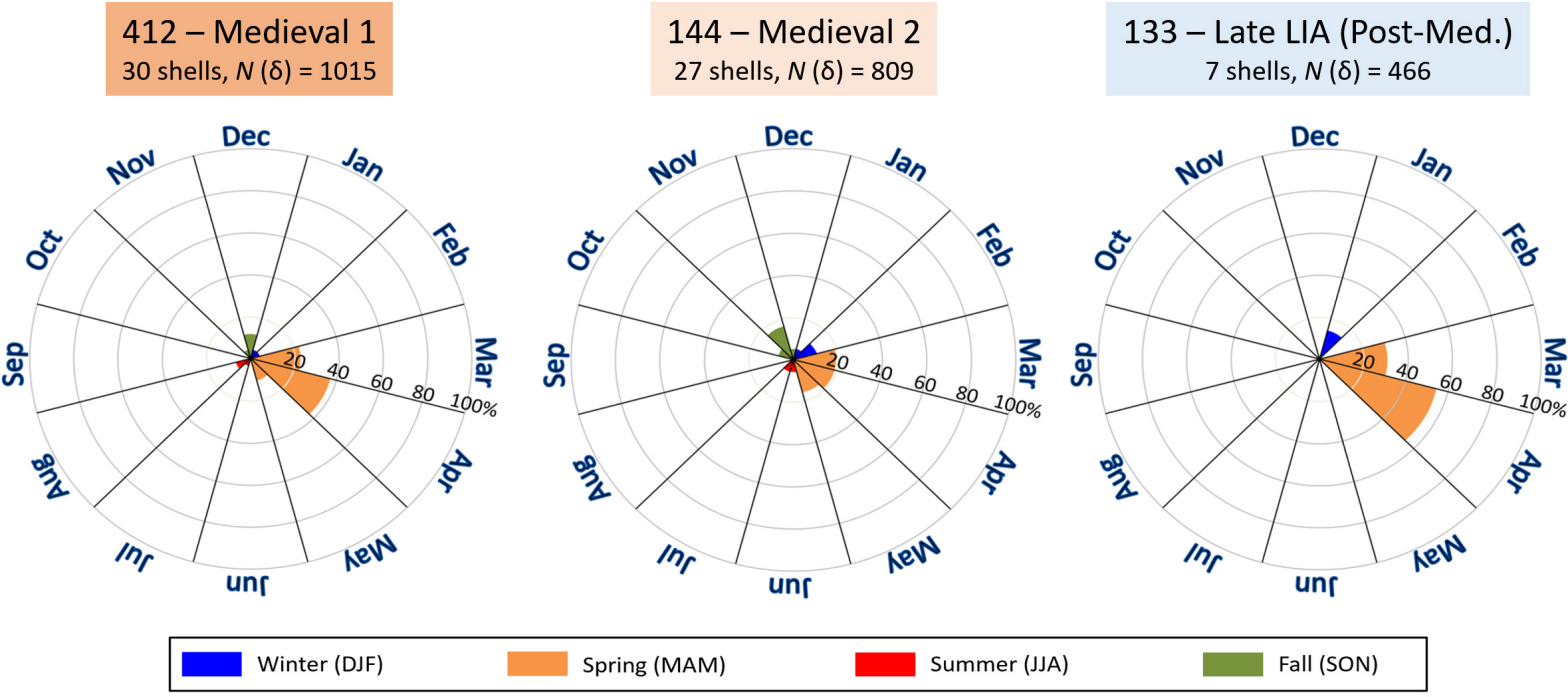
Season-of-collection data of *Littorina littorea* specimens from Skaill Farm determined to the nearest month based on temporally aligned *δ*^18^*O*_shell_-derived temperature (T*δ*^18^*O*) data. (left) Medieval 1, (middle) Medieval 2, (right) Post-medieval (late LIA). Lower case Greek delta refers to shell oxygen isotope data. See also table 1. DJF = December to February; MAM = March to May; JJA = June to August; SON = September to November.

Reconstruction of the season of collection (figure 4) was done by comparing the T*δ*^18^*O* shape and amplitude of the last year of growth with the seasonal temperature variation in previous years and/or the modern long-term seasonal temperature curve. It was then possible to date the last sample from the aperture to the nearest month or so, based on the missing part of the T*δ*^18^*O* curve relative to the previous and following winter or summer (see also electronic supplementary material).

### (f) Resampling

**Figure 5.**
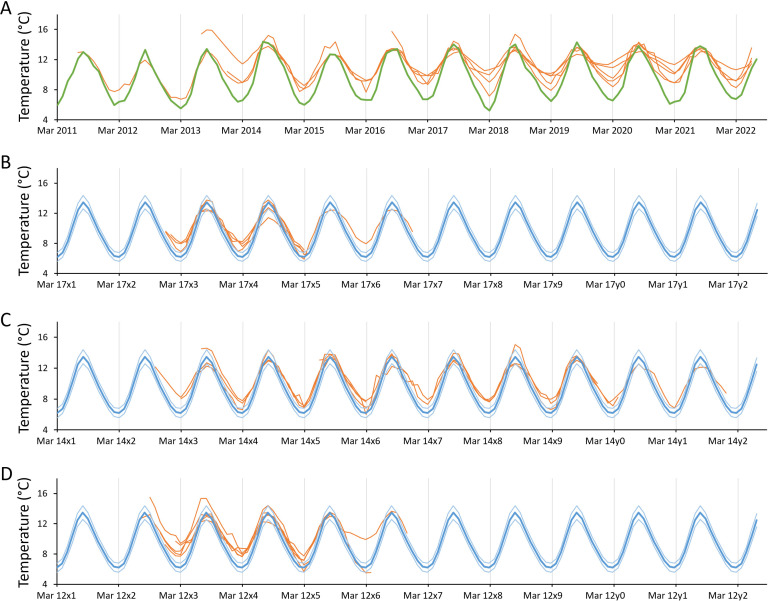
Monthly resampled, temporally aligned *δ*^18^*O*_shell_-derived temperature (T*δ*^18^*O*) data of *Littorina littorea* from Skaill Farm. By contrast to data depicted in electronic supplementary material, figure S5, each data point now represents the same amount of time (= one month) and can thus be directly compared with any other data point of any specimen depicted. (A) Modern specimens collected alive on 14 July 2022, (B–D) archaeological specimens from the (B) Late Little Ice Age, (C) Medieval 2 and (D) Medieval 1. See caption of electronic supplementary material, figure S5 for further details.

One major goal of the present study was to compare the T*δ*^18^*O* curves with each other, specifically summers and winters of different years and specimens from the same and different time intervals, in order to test the reproducibility of temperature reconstructions from contemporaneous specimens and determine the climate state in the studied time intervals. However, each sample taken from the shells represented different amounts of time, with the consequence that isotope data from years with higher temporal resolution would show larger amplitudes than samples from slower-growing annual increments (e.g. later during life). It was thus necessary to compute a new set of data with the same temporal resolution, here monthly (electronic supplementary material, figure S10). To do so, linear imputation was employed to compute missing T*δ*^18^*O* values between centres of sample spots and generate an uninterrupted T*δ*^18^*O* series with daily resolution. This new time-series was then resampled to obtain monthly T*δ*^18^*O* data (figure 5). Note that the only years considered were those for which at least one isotope sample existed for each month during the warmest (July–September) and coldest parts of the year (January/February–March/April).

## 3. Results

### (a) Age and shell size

Stable oxygen isotope curves of studied shells showed distinct seasonal oscillations (figure 5, electronic supplementary material, S6). The largest number of such cycles, i.e. the longest lifespan (11 years) was observed in the biggest live-collected specimen (A2), with an apex-to-aperture length of 36.4 mm and an unrolled length of 14.3 cm (figure 3A). All 15 ontogenetically sampled specimens from the kitchen middens were shorter-lived (average age: 3.4 ± 1.4 years) and smaller (apex-aperture: 27.8 ± 3.8 mm; unrolled: 10.7 ± 1.6 cm) than modern specimens (average age: 7.7 ± 2.7 years; apex-aperture: 12.9 ± 4.0 mm; unrolled: 12.9 ± 1.4 cm) (figure 3B,C). This was especially true for the periwinkles recovered from horizon 133 (late LIA) that only reached an age of one to four years and an average apex-aperture length of 25.6 mm (unrolled length: 9.8 cm). Non-parametric tests (Wilcoxon) partly confirmed these observations, i.e. only half of the pairs were statistically significantly different (figure 3A–C; table 2).

**Table 2.** Pairwise, non-parametric statistical comparison (Wilcoxon test) of *Littorina littorea* data depicted in figure 3. Statistically significantly different means are listed in bold font. Numbers in parentheses denote numbers of specimens used in comparison; first value refers to upper row of header, second value to second row of header. Note, figure 3A–C comprise only specimens used for palaeoclimate analysis, i.e. from which ontogenetic isotope profiles were generated. Figure 3D includes data from some additional archaeological specimens (*) that were predominantly used to determine the season of collection (compare table 1).

figure		modern vs.	modern vs.	modern vs.	late LIA vs.	late LIA vs.	Medieval 2 vs.
		late LIA	Medieval 2	Medieval 1	Medieval 2	Medieval 1	Medieval 1
7A	lifespan	**0.0163 (5, 5)**	0.0758 (5, 5)	**0.0163 (5, 5)**	**0.0283 (5, 5)**	0.2506 (5, 5)	0.0758 (5, 5)
7B	apex-to-aperture length	0.0758 (5, 5)	0.6015 (5, 5)	0.1172 (5, 5)	**0.0472 (5, 5)**	0.3472 (5, 5)	0.1745 (5, 5)
7C	unrolled length	**0.0163 (5, 5)**	0.1172 (5, 5)	0.0758 (5, 5)	**0.0283 (5, 5)**	0.7540 (5, 5)	0.1745 (5, 5)
7D	apex-to-aperture length*	**0.0500 (5, 10)**	0.4642 (5, 8)	0.0512 (5, 23)	**0.0263 (10, 8)**	0.1960 (10, 23)	**0.0367 (8, 23)**

Results for the apex–aperture length comparison remained nearly identical when the data from 26 additional archaeological shells were included (figure 3D; electronic supplementary material, table 1+2). Although modern shells attained larger sizes than archaeological shells, their mean sizes were still not statistically significantly different from Medieval 2 (context 144) and Medieval 1 (context 412) specimens, but they did differ significantly from snails of Post-medieval date (context 133, late LIA). The average size of late LIA specimens was still significantly larger than that of Medieval 2 shells, and the latter were significantly larger than such from Medieval 1 (figure 3D; table 2).

### (b) Timing of annual growth line formation

The highest *δ*^18^*O*_shell_ values of each seasonal cycle reflecting the coldest part of the year were typically recorded shortly after the annual growth line (electronic supplementary material). This was especially evident in specimens that died during late winter and underwent high-resolution (= apertural) micromilling. For example, in specimens 133-D1, 2, 3 and 5 as well as 144-D1 (electronic supplementary material, figure S11), several samples taken by apertural micromilling after the annual growth line revealed lower *δ*^18^*O*_shell_ values than samples taken before the growth line (electronic supplementary material). The same was found in almost all OSS-micromilled samples, e.g. in the vicinity of the second ‘winter’ line (counted from the apex) in specimen 133-D5 and most years of specimen 144-D1 (electronic supplementary material, figure S11). By contrast, the coldest part of year four in specimen 144-D1 was represented by several OSS micromilled samples with nearly identical *δ*^18^*O*_shell_ values. Here, the annual growth line was positioned just in the middle of these samples, i.e. its formation may have occurred when the lowest temperature prevailed (electronic supplementary material, figure S11).

### (c) Temporal alignment of the shell record, oxygen isotope offset

As shown in electronic supplementary material, figure S6, once aligned to the sinusoidal shape of the temperature curve, the best fit between reconstructed and observed summer temperatures was achieved when a constant offset was applied to *δ*^18^*O*_shell_ values, i.e. −1.30 ‰ in case of specimens A1, 2 and 5, and merely −0.95 and −1.20‰ in case of specimens A3 and A4, respectively (electronic supplementary material, figure S12A+B). Note that even with this smaller offset, Tδ^18^O during the first year of life of specimen A4 overestimated actual temperatures by more than 2.5°C in summer and 5°C in winter, while the −1.20‰ offset worked well for summers in the following years (electronic supplementary material, figure S12B). Interestingly, the two specimens that required a smaller isotopic offset showed strong high-frequency Tδ^18^O variance during spring to fall at age 3 years, with temperature oscillations of as much as 4°C (electronic supplementary material, figure S12A+B). Note, in the preceding 2 years, the time-averaging of the samples was larger than during years 3 to 5.

Hinging the Tδ^18^O data at the instrumental summer temperatures and selecting the *δ*^18^*O*_shell_ offset accordingly seemed justified because the isotope data density (electronic supplementary material, figure S6) and thus temporal resolution (and shell growth rate; electronic supplementary material, figure S7) were highest in the warm season. In addition, according to field and laboratory observations, the common periwinkle is much less active during the cold season [40], likely resulting in a larger time-averaging of winter samples. It should be added that the temporal alignment of the shell record as well as T*δ*^18^*O* values remained largely unchanged if the seasonal variation of *δ*^18^*O*_water_ was taken into consideration (electronic supplementary material, figure S12C).

### (d) Winter temperatures

During lifetime, reconstructed winter temperatures increased progressively, while summer Tδ^18^O fluctuated from year to year without showing a directed trend (electronic supplementary material, figure S6). As a consequence, seasonal amplitudes became gradually attenuated with increasing ontogenetic age. The offset of reconstructed from measured winter temperatures increased, on average, *ca* 0.3°C per year (electronic supplementary material). Notably, in modern specimens, winter temperatures were always overestimated, even during age 1 (on average, by 2.2 ± 0.8°C; propagated error: 1σ variance of mean winter Tδ^18^O = 0.32°C, 1*σ* analytical uncertainty = ± 0.12‰ and 1*σ* variance of *δ*^18^*O*_water_ = ± 0.15‰ corresponding to 0.46 and 0.57°C, respectively) (figure 5A, electronic supplementary material, S6A), provided that the above-listed *δ*^18^*O*_water_ offsets were applied. The winter temperature bias was negatively correlated with shell growth rate (electronic supplementary material), i.e. a reduction in annual increment width by 9.5 mm per year resulted in 1°C higher Tδ^18^O. The only exception was the last winter of specimen A2 (electronic supplementary material, figure S12C). Based on the age of this specimen at that time (11 years), an offset of *ca* 5°C would have been expected. However, the actual offset was merely 2.5 ± 0.8°C. As mentioned in §2, the last annual growth increment of that specimen was sampled by the apertural micromilling technique instead of OSS micromilling.

### (e) Climate variability through time

**Figure 6.**
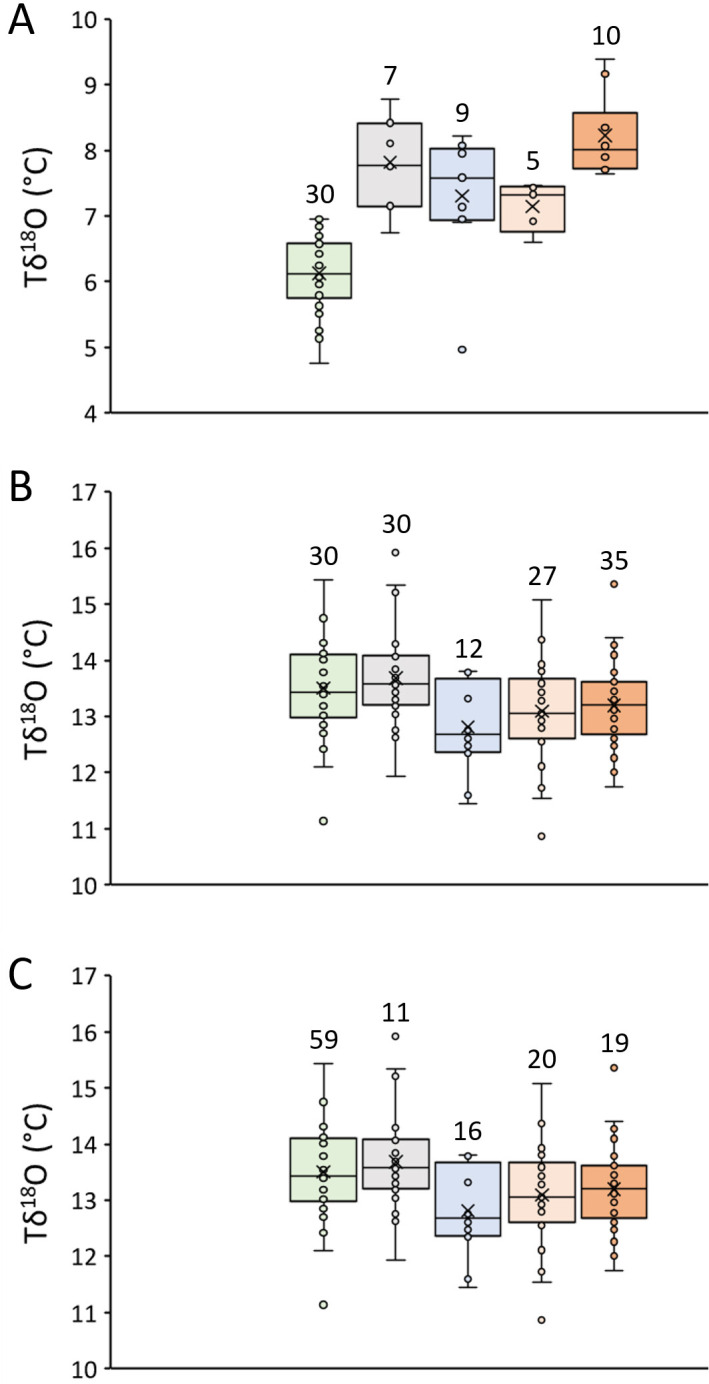
Robustness of temperature reconstructions based on *δ*^18^*O*_shell_ data of *Littorina littorea*. Winter temperatures (A) and the seasonal temperature amplitude (C) were significantly overestimated by modern specimens, whereas summer temperatures (B) were nearly perfectly resembled. For pairwise statistical comparisons, see table 3. Numbers above boxplots refer to numbers of winters, summers and seasonal temperature ranges. Colour codes as in the caption of figure 3. Light green stands for modern temperatures during the 30-year interval of 1992/3−2022.

To assess how well modern shells reflected the current climate state and how palaeoclimate deviated from the present conditions, winter and summer Tδ^18^O as well as seasonal Tδ^18^O amplitudes were compared with the average seasonal temperature extremes during the past 30 years prior to shell collection (1992/3−2022; = climatic normal) (figure 6; table 3). As shown in figure 6B and table 3, modern shell-derived summer temperatures (*n* = 30) were statistically indistinguishable from instrumental temperatures of the reference period (13.68 versus 13.50°C, respectively). The same was true for the Medieval 1 (*n* = 35; 13.19°C) and Medieval 2 summers (*n* = 27; 13.09°C), respectively. Only during the late LIA, with 12.81°C, was the warm season significantly colder than today (*n* = 12).

**Table 3.** Pairwise, non-parametric statistical comparison (Wilcoxon test) of palaeoclimate data reconstructed from *δ*^18^*O*_shell_ data of *Littorina littorea* depicted in figure 6. Statistically significantly different means are list in bold font. Numbers in parentheses denote number of specimens used in comparison; first value refers to upper row of header, second value to second row of header.

	30 yr SST				modern			late LIA		Medieval 2
	modern	late LIA	Medieval 2	Medieval 1	late LIA	Medieval 2	Medieval 1	Medieval 2	Medieval 1	Medieval 1
winter	**<0.0001 (30, 7)**	**0.0006 (30, 9)**	**0.0019 (30, 5)**	**<0.0001 (30, 10)**	0.3146 (7, 9)	0.0882 (7, 5)	0.4945 (7, 10)	0.3173 (9, 5)	**0.5000** **(9, 10)**	**0.0220** **(5, 10)**
summer	0.4824 (30, 30)	**0.0249 (30, 12)**	0.0949 (30, 27)	0.0959 (30, 35)	**0.0063 (30, 12)**	**0.0058 (30, 27)**	**0.0049 (30, 35)**	0.2938 (12, 27)	0.2939 (12, 35)	0.8036 (27, 35)
seasonality	**0.0002 (59, 11)**	**<0.0001 (59, 16)**	**<0.0001 (59, 20)**	**<0.0001 (59, 19)**	0.2774 (11, 16)	0.9178 (11, 20)	0.0582 (11, 19)	0.1564 (16, 20)	0.1360 (16, 19)	**0.0073** **(20, 19)**

The mean winter temperature of the past 30 years was significantly overestimated by modern shells by 1.69°C (*n* = 7; note, the only winters considered during ages 1 and 2 were those during which at least one Tδ^18^O value was available from each month between January and April) and the average seasonal temperature range was nearly 1.4°C lower than indicated by observational data (*n* = 11) (figure 6A,C; table 3). Winters during all 3 studied time intervals of the past were likewise significantly warmer than the modern climatic normal (*n* = 9, 5 and 10 for late LIA, Medieval 2 and Medieval 1, respectively) (figure 6A,C; table 3). Medieval 1 winters seem to have been *ca* 0.4°C warmer than today, but winters remained 0.7 and 0.5°C colder during the slightly later Medieval 2 and Post-medieval, respectively, reflecting a temperature shift starting in the later 14th to early 15th centuries and spanning the LIA. In the later Medieval 2 times, the mean seasonal temperature amplitude seems to have been nearly identical to today, whereas the temperature spread during winter and summer was 0.3 and 0.7°C larger during the late LIA and Medieval times, respectively (*n* = 16, 20 and 19 for late LIA, Medieval 2 and Medieval 1, respectively).

### (f) Shell growth rate

Cumulative annual shell growth curves revealed only small differences between the four studied time intervals (electronic supplementary material, figure S8). During Post-medieval and Medieval 2 times, shells grew, on average, up to *ca* 10% slower than at present and during Medieval 1 times, reflecting a decrease in growth rate starting around the later 14th to early 15th century that remains consistent through the LIA. As illustrated by the relative seasonal growth curve of all specimens and time intervals, slowest and fastest shell production did not fall together with seasonal temperature extremes (electronic supplementary material, figure S7). Minimum growth was observed, on average, *ca* one month before the coldest month (Feb/Mar), and maximum growth (= 0.55% of the annual growth increment width formed per day), on average, *ca* one month before the summer temperature maximum.

### (g) Shell structure

Cross-sections through the aperture revealed two distinct shell layers (electronic supplementary material, figure S2): a thick OSL (near the aperture *ca* 30 to 3.5 mm) and a thin ISL (near the aperture *ca* 300–500 µm). The ISL (aragonite) turned black after treatment with Feigl solution for a few minutes, while the OSL (low-Mg calcite) assumed only a light grey tint (figure 2). The OSL was further subdivided into an outer, pigment-rich (olive-brownish colour; *ca* 1 mm thick) and an inner pigment-poor portion (white) (electronic supplementary material, figure S2). Importantly, in winter, the ISL followed the shape of the growth pattern and occasionally reached the OSS (figure 2). In that case, it would be impossible to obtain powder from the shell near winter lines without an admixture of aragonite from the ISL.

Annual growth lines could not only be easily identified by a constriction on the OSS, but also by the much brighter colour than the rest of the annual increment, which had an olive-brownish tint, at least in more recent specimens (electronic supplementary material, figures S2, S4, S11). Growth lines separating growth increments were oriented subparallel to the sigmoidal shape of the lip of the shell and approached the OSS with an angle of about 45° (electronic supplementary material, figure S2). At higher magnification, bundles of broad and narrow microgrowth increments were observed (electronic supplementary material, figure S1) that resembled fortnight cycles, also supported by a rough agreement of the number of these µm-scale growth patterns (*ca* 14) with the expected number of lunar days per fortnight cycle.

### (h) Season of collection

In all three archaeological time intervals, most shells were collected in spring (52–86%), specifically April (19–57%) (figure 4). During the Medieval 1 and Medieval 2 phases, 30 and 48% of the specimens, respectively, were harvested in the three other seasons, specifically fall (up to 22%). During the late LIA, 14% of the specimens died in January, but the rest in March/April—precisely when less food would be available.

## 4. Discussion

### (a) *Littorina littorea—*a recorder of summer water temperature

As demonstrated here, summer temperatures can be robustly estimated from *δ*^18^*O*_shell_ curves of *L. littorea* as long as the *δ*^18^*O*_water_ value is known and a constant *δ*^18^*O*_shell_ offset from expected equilibrium is considered (figure 6B; table 3). With an average *δ*^18^*O*_water_ value of −0.19 ± 0.15‰ (2022; 1 *σ* error translates into a temperature uncertainty of *ca* 0.7°C) and a *δ*^18^*O*_shell_ offset of −0.95 to −1.30‰, inter-annual changes of summer temperature were well reflected by the *δ*^18^*O*_shell_ data, specifically if values from different contemporaneous specimens were combined to reduce individual differences. The average summer temperature during the 30-year interval (1992−2021) prior to shell collection was overestimated by only 0.18°C, although isotope data from only 10 consecutive years (2012−2021) were available from modern shells. With additional specimens covering the 20 years before 2012 it may have been possible to further reduce this difference and meet the requirement of a true climate reconstruction, i.e. covering >30 consecutive years [41,42].

Aside from *δ*^18^*O*_water_ fluctuations, a temperature bias can also result from individual departures from isotopic equilibrium (= vital effects). While four out of five live-collected specimens showed a *δ*^18^*O*_shell_ offset from expected equilibrium of −1.30 to −1.20‰, one specimen required a shift of merely −0.95‰. If such a difference remained unnoticed (which is unavoidable when fossil shells are used) and the higher offset (−1.30‰) was applied instead, actual temperatures were underestimated by that particular specimen by *ca* 1.3 to 1.5°C. With five specimens per calendar year (as in the present study), the temperature bias can then be limited to about +0.3°C (assuming similar differences in vital effects to those observed in modern shells). A higher replication could further reduce the temperature bias introduced by specimen-specific offsets from isotopic equilibrium.

Constant isotopic disequilibria have been reported before from several marine gastropods [52–54] and a range of possible explanations discussed [50]. The more surprising finding in the present study was that departures from isotopic equilibrium varied between contemporaneous specimens from the same site and—in two specimens—through lifetime (electronic supplementary material, figure S12A+B). For example, the isotope data of specimen A3 deviated by only −0.95‰ from expected equilibrium, while the others exhibited 0.25 to 0.35‰ larger offsets. This particular snail may have lived closer to the coast where freshwater influx or groundwater infiltration was stronger than in the mid- and low intertidal zone (where most of the remaining specimens likely dwelled throughout their lifetime), leading to lower *δ*^18^*O*_water_ values. Alternatively, the *δ*^18^*O*_water_ signature may have remained largely invariant across the shelf, but vital effects varied between specimens. In specimens A3 and A4, isotopic disequilibria gradually diminished during the first 3 years of life, possibly indicating wave-induced dislocations from the high to the low intertidal, i.e. toward isotopically more stable sites. Support for this assumption comes from a larger *δ*^18^*O*_shell_ variance (raw, non-resampled data) during youth—specifically year 3—than later during life and compared with other specimens that were sampled with the same temporal resolution. During youth, these two specimens may have been exposed to large short-term *δ*^18^*O*_water_ swings caused, for instance, by freshwater influx and precipitation, as well as by evaporation (in tide pools). Judging from results of behavioural studies [40], it seems unlikely that the snails actively moved to a new homebase during youth because periwinkles are known to remain connected to the place where they grew up. *L. littorea* typically returns to its homebase after feeding excursions, but does not find their way back after a major dislocation [40], e.g. by a storm event.

Individual isotopic disequilibria (vital effects) and gradual *δ*^18^*O*_water_ shifts owing to movement in an isotopically variable habitat can pose major challenges for climate reconstructions. One way to mitigate such challenges is to increase the number of studied specimens and years and preclude specimens or individual years with large, short-term isotopic swings. Furthermore, a meticulous sclerochronological analysis of tidal shell growth patterns [34] (electronic supplementary material, figure S1) can help to determine the position at which the specimens lived within the intertidal zone during different stages of life. Specimens from the high intertidal, for instance, could then be precluded from isotopic analyses. As outlined by Ohno [55] and further explained in Schöne [56], the widths and number of (semi)diurnal growth increments decrease from the low to the high intertidal because the time available for shell growth during each tidal submersion decreases toward the coast and during neap tides and the water does not reach the high intertidal on all days, resulting in missing days in the shell record. The strong link between the tidal cycles and shell growth patterns of *L. littorea* has been experimentally verified by Ekaratne & Crisp [34,57]. They also demonstrated that microgrowth lines are formed during low tide (= tidal emersion, aerial exposure; [57]) as in other intertidal gastropods [58] and bivalves [59–61]. Hence, the technique introduced by Ohno [55] for bivalves can also be applied to intertidal gastropods to determine their feeding grounds on the tide flats relative to the coastline.

### (b) Winter temperatures

By contrast to summer temperatures, actual monthly winter temperatures were always overestimated by *δ*^18^*O*_shell_, even in fast-growing, juvenile shell portions (on average, by 2.2 ± 0.8°C) as well as in sections sampled with higher resolution, i.e. by apertural micromilling (+2.5 ± 0.8°C in year 11 of specimen A2) (figures 5 and 6 and electronic supplementary material, S6, S12). Winters in respective shell portions were often represented by several samples with similarly high *δ*^18^*O*_shell_ values, suggesting that shells grew uninterruptedly during the cold season, and in case of juveniles (age 2−3 years), still at a moderate rate providing better than monthly resolution (electronic supplementary material, figure S11; Supplements). This assumption is supported by sclerochronological data from Ekaratne & Crisp [34], who observed continuous shell growth in this species during winter, even when air temperatures remained below the freezing point (up to −6°C) for a week. Although the common periwinkle reportedly becomes largely inactive below 8°C [40], this apparently does not affect shell growth.

Strong time-averaging can thus not explain the overestimation of winter temperatures in juveniles and shell portions sampled with monthly and better resolution. More likely, the *δ*^18^*O*_water_ value during winter was lower than suggested by the biweekly measurements in 2022 (electronic supplementary material), and neither the annual average nor the mean winter *δ*^18^*O*_water_ of 2022 (which was nearly identical to the annual mean) were representative for shells that grew during the coldest part of winter 2022, and much less for prior years. It should also be considered that *δ*^18^*O*_water_ in other years was reconstructed from salinity, which in turn was only measured once per week and exhibited the strongest variability during the cold season (electronic supplementary material). Furthermore, temperature, salinity and *δ*^18^*O*_water_ were only measured at some distance from Skaill Farm (figure 1) and may thus not be representative of local conditions. Hence, actual *δ*^18^*O*_water_ values could have been lower than assumed, which would explain the discrepancies between measured and reconstructed temperatures in juveniles and shell portions sampled with higher resolution. Future studies should explore the possibility of verifying reconstructed water temperatures through carbonate clumped isotope analysis (*Δ*_47_ values) and potentially use such data in conjunction with *δ*^18^*O*_shell_ values to quantify salinity and *δ*^18^*O*_water_ signatures.

The gradually rising winter temperature through ontogeny (figure 5, electronic supplementary material, S6, S12), in turn, can be easily explained by increasing time-averaging of respective shell samples. With OSS micromilling it was often impossible to obtain more than one sample for the entire cold season (electronic supplementary material, figures S6, S12). However, a change in the timing and rate of seasonal shell growth seems unlikely, because otherwise the seasonal temperature amplitude determined in the previous to the penultimate year of life in the oldest specimen (A2) would not have been identical to that measured during youth (electronic supplementary material, figure S12C).

Stronger time-averaging in ontogenetically older shell portions laid down during winter and uncertainties regarding the winter *δ*^18^*O*_water_ value potentially have implications for season-of-collection studies. Unless a higher-resolution sampling technique is applied and independent ways are established to determine the salinity in winter (from which *δ*^18^*O*_water_ signatures can be computed), estimates of the season of harvest between approximately January and April will remain elusive.

In the current paper, we were unable to determine the lower shell growth temperature limit of the common periwinkle. The lowest monthly winter temperatures of 5.25 °C (2018) and 5.50 °C (2013) (electronic supplementary material) seem to have been higher than the critical threshold for biomineralization of this species (see §4). Following Burman & Schmitz [36], *L. littorea* shuts down shell growth once temperatures drop below 3.7 ± 1.0°C. This lower temperature threshold was deduced from the highest *δ*^18^*O*_shell_ data determined in several modern, live-collected specimens. However, their entire study is based on an incorrect palaeotemperature equation that leads to an overestimation of summer temperatures by nearly 1°C (based on which a *δ*^18^*O*_shell_ offset of −2‰ was computed) and an overestimation of winter temperatures by *ca* 1.8°C, because they fitted a linear to the *δ*^18^*O*_shell_ vs temperature data obtained by the fractionation relationship by Friedman & O’Neill [51]. Actually, the oxygen isotope palaeothermometry equation by Hays & Grossman [49], which was used in the present study, is the quadratic approximation of the expression by Friedman & O’Neill [51] that correctly considers the non-linear relationship between *δ*^18^*O*_shell_ and temperature.

Shortcomings of the method by Burman & Schmitz [36] could have implications for studies build upon them. For example, Lewis *et al.* [37] recently displroved the hypothesis that the malacological change observed in classical Danish shell middens at the Mesolithic–Neolithic transition (at *ca* 5900 cal. yr BCE) were caused by a shift in salinity. The latter was computed from highest winter *δ*^18^*O*_shell_ value given by the local freshwater mixing line, as well as the linear palaeotemperature equation presented in Burman & Schmitz [36]. Results of this study should be verified and data recalculated. Presumably, the overall finding will remain unchanged, but absolute salinity will be different.

### (c) Seasonal shell growth

Seasonal growth curves from all four studied time intervals suggested that neither slowest nor fastest shell growth was directly linked to temperature extremes (electronic supplementary material, figure S7). For example, the annual growth line, reflecting particularly slow growth (not necessarily cessation, as this would remain undocumented anyway: no shell growth means no growth line), was typically formed *ca* 1 month before the seasonal temperature minimum, and fastest shell growth was observed *ca* 1 month before the summer temperature maximum (electronic supplementary material, figures S7, S11). These findings largely agree with observations by Ekaratne & Crisp [34], according to which shell growth was highest during algal blooms in May and September and slowest in January/February when food quantity and quality were low. Notwithstanding the relevance of temperature for metabolic rates of poikilotherms, food availability seems to have driven shell growth even more strongly than temperature alone.

### (d) Pitfalls during sampling

A surprising finding herein was that the aragonitic inner shell layer occasionally stretches up to the OSS during the coldest part of winter (figure 2). It can therefore be difficult or impossible to obtain pure low-Mg calcite samples from shell portions near ‘winter’ lines. If the admixture of aragonite remains unnoticed or is ignored and the palaeotemperature equation by Hays & Grossman [49] is used, winter temperatures will be underestimated by up to *ca* 3.2°C (assuming the highest *δ*^18^*O*_shell_ value of the present study = +3.36 ‰, *δ*^18^*O*_water_ = +1.10 ‰, 100% aragonite). Very shallow OSS micromilling, as done herein, can significantly reduce or entirely eliminate the danger of aragonite contamination. It is therefore strongly recommended to study the internal shell structure and mineralogy (e.g. with Feigl solution [46,47]; figure 2) in cross-sectioned shells prior to isotope sampling and to devise a suitable sampling strategy. It is noteworthy that the presence of aragonite indicates small/non-existent diagenetic overprint; the environmental data encoded in the chemical properties of the shell are then likely to be pristinely preserved.

### (e) Palaeoclimate data and information on past human societies

As outlined above, owing to uncertainties of the actual *δ*^18^*O*_water_ signatures during winter, useful information on past climatic conditions can only be deduced from summer *δ*^18^*O*_shell_ data of *L. littorea* (figure 6). In comparison with modern times, summer water temperature near Skaill Farm was statistically significantly lower (by *ca* 0.7°C) during the late LIA, whereas the two Medieval contexts sampled were statistically indistinguishable from today. However, the limited number of studied annual increments (*n* = 12) and shells (*n* = 6) from context 133 renders robust inferences on the true climatic state during the LIA difficult. We did not anticipate that specimens from that time interval were so short-lived; this has implications for our understanding of human dietary pressures on the foreshore.

Actually, the lifespan of all archaeological specimens remained below that of modern periwinkles (figure 3A; table 2). One possible explanation—for which, however, no ethnographic evidence exists—is that people living at Skaill Farm preferentially collected small, young snails, perhaps owing to their better taste. Alternatively, large old-grown specimens were already exhausted owing to overharvesting. However, ontogenetically older specimens were not found in any of the studied archaeological layers, so the overharvesting hypothesis should be dismissed. Other than as implied by size data depicted in figure 3, ongoing metrical analysis of 4975 periwinkles found in the same three contexts analysed here indicate no significant differences in height underlining the necessity to study a large number of specimens.

An initial analysis of the season of collection suggested that periwinkles were preferentially collected during early spring, specifically during the late LIA (figure 4). This potentially indicates that these snails served as a starvation food at times when winter stocks were running low. During late spring and summer, snails may have been collected as a food supplement. However, soft tissues of the snails may also have been used as bait. Future ultrastructural analysis of the shells can shed light on the question of whether the snails were boiled and consumed, or otherwise used raw (note, shells did not reveal any signs of burning in fire). Cooking helps to extract the meat from the shells and is a typical practice when shellfish are consumed [62]. If the snails were boiled, the shell ultrastructure may show minute changes in comparison with uncooked specimens [63]. To provide a comprehensive interpretation of why the snails were collected and to contextualize the periwinkles in a broader subsistence base, future studies should use additional faunal information from the studied site.

## 5. Summary and conclusions

Results of the present study led the (refined) groundwork for subsequent, more detailed shell midden analysis at Skaill Farm and other localities where kitchen waste deposits contain well-preserved shells of the common periwinkle, *L. littorea*. As demonstrated, between ambient temperatures of 5.3 and 14.4°C (monthly SST averages), shells grow throughout the year, although they are retarded in early winter before the seasonal temperature minimum, i.e. at the time when the annual growth line is laid down. Fastest growth occurs in *ca* mid-July, i.e. one month before the seasonal temperature maximum.

Like many other intertidal gastropods, this species forms its shell with a constant *δ*^18^*O*_shell_ offset of −1.30‰ from expected equilibrium. If this offset is considered and the *δ*^18^*O*_water_ signature during growth is known (or can be estimated), *δ*^18^*O*_shell_ can provide robust summer temperature estimates. Here, the modern 30-year summer temperature average was overestimated by merely 0.18°C. Reconstruction of the winter temperature is far more challenging and should be limited to juvenile shell portions (age 1 and 2). Still, *δ*^18^*O*-based winter temperature estimates require high-resolution sampling because growth rates are slower in the cold season, leading to larger time-averaging. Prior to isotope sampling, the growth pattern geometry and distribution of CaCO_3_ polymorphs need to be assessed in cross-sections. In general, we recommend very shallow OSS micromilling—perhaps with even finer drill bits than those used here—to avoid/limit contamination of the shell powder samples with aragonite from the inner shell layer. Apertural micromilling can provide temporally higher-resolved data, but this is more susceptible to aragonite admixture. If this contamination remains unnoticed, the use of the palaeothermometry equation for low-Mg calcite leads to an underestimation of winter temperature by up to 3.2°C.

To account for potential individual differences in isotopic disequilibrium, replication is essential, i.e. several contemporaneous specimens should be analysed. To properly determine the (palaeo)climatic state, a large number of annual increments were required, ideally more than 30 consecutive years. Although we were unable to obtain 30-year data ranges from each stratigraphic horizon, we still generated a solid baseline to assess the climatic state for three of the four studied time intervals, i.e. the two phases of Medieval occupation and the modern era at Skaill Farm, Orkney. From each of these stratigraphic levels, ≥30 annual increments were isotopically characterized. Owing to the short lifespan of the common periwinkle (here max. age = 12 years), it will remain largely impossible to obtain an environmental record of 30 consecutive annual increments. With modern specimens this may be achieved in future studies by using specimens from museum collections with known dates of death. In case of archaeological specimens, this criterion is nearly impossible to meet.

The season of collection of archaeological specimens can be determined to the nearest month by comparison of the oxygen isotope-derived temperature data with the local long-term seasonal temperature curve. Such reconstructions are possible in both juveniles and adults, because the timing and rate of seasonal shell growth remain invariant throughout the lifetime. Evidently, in old-grown specimens, a higher sampling resolution is required than in young individuals. Best results are achieved if the last year at the aperture is sampled. Following initial results, inhabitants of Skaill Farm, Rousay, Orkney, likely collected periwinkles as a starvation food once the winter supplies were running low. A preference for small, young individuals seems to have prevailed. Future studies should further explore the possibility of refining the season of harvest by using tidal shell growth patterns. This technique may also be exploited as a faster alternative to isotope-based season-of-collection analyses and help to generate larger datasets.

## Data Availability

The datasets supporting this article have been uploaded as part of the electronic supplementary material [64].
